# Early prediction of autoimmune (type 1) diabetes

**DOI:** 10.1007/s00125-017-4308-1

**Published:** 2017-05-26

**Authors:** Simon E. Regnell, Åke Lernmark

**Affiliations:** Department of Clinical Sciences, Lund University/CRC, Skåne University Hospital, Jan Waldenströms gata 35, SE-20502 Malmö, Sweden

**Keywords:** Autoimmunity, Beta cells, diabetes mellitus, Glutamic acid decarboxylase autoantibodies, HLA, Insulin autoantibodies, Insulin secretion, Insulinoma-associated antingen-2 autoantibodies, Next-generation sequencing, Review, Type 1 diabetes, ZnT8 autoantibodies

## Abstract

Underlying type 1 diabetes is a genetic aetiology dominated by the influence of specific HLA haplotypes involving primarily the class II *DR-DQ* region. In genetically predisposed children with the *DR4-DQ8* haplotype, exogenous factors, yet to be identified, are thought to trigger an autoimmune reaction against insulin, signalled by insulin autoantibodies as the first autoantibody to appear. In children with the *DR3-DQ2* haplotype, the triggering reaction is primarily against GAD signalled by GAD autoantibodies (GADA) as the first-appearing autoantibody. The incidence rate of insulin autoantibodies as the first-appearing autoantibody peaks during the first years of life and declines thereafter. The incidence rate of GADA as the first-appearing autoantibody peaks later but does not decline. The first autoantibody may variably be followed, in an apparently non-HLA-associated pathogenesis, by a second, third or fourth autoantibody. Although not all persons with a single type of autoantibody progress to diabetes, the presence of multiple autoantibodies seems invariably to be followed by loss of functional beta cell mass and eventually by dysglycaemia and symptoms. Infiltration of mononuclear cells in and around the islets appears to be a late phenomenon appearing in the multiple-autoantibody-positive with dysglycaemia. As our understanding of the aetiology and pathogenesis of type 1 diabetes advances, the improved capability for early prediction should guide new strategies for the prevention of type 1 diabetes.

**Electronic supplementary material:**

The online version of this article (doi:10.1007/s00125-017-4308-1) contains a slideset of the figures for download, which is available to authorised users.

## Introduction

### Predicting and staging autoimmune (type 1) diabetes

Type 1 diabetes is a chronic disease in which genetic predisposition, coupled with environmental influences predominantly early in life, induces pancreatic beta cell autoimmunity eventually resulting in both loss of function and destruction. The loss of beta cells leads to gradually diminishing insulin production, loss of blood sugar control and subsequent dependence on exogenous insulin administration; risk of developing long-term complications ensues.

**Fig. 1:**
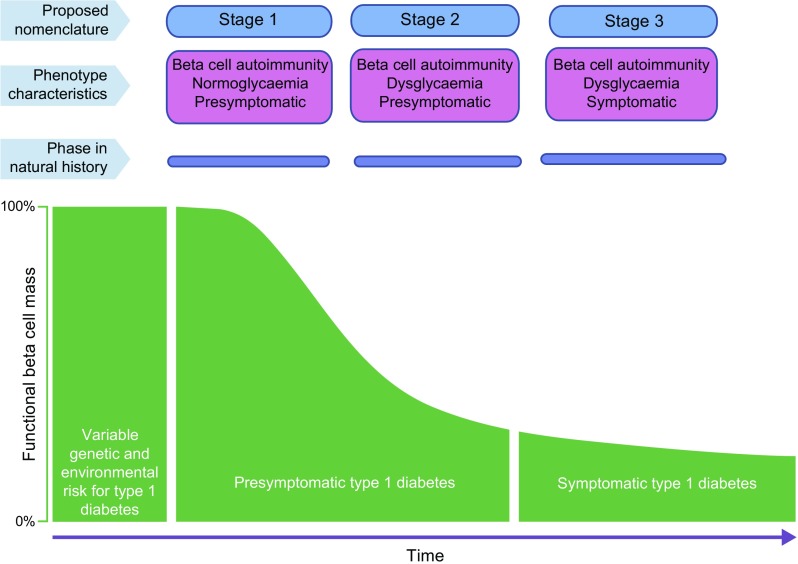
Proposed staging of type 1 diabetes. The aetiology is represented by a variable genetic and environmental risk. The pathogenesis is represented by three stages. In stage 1, beta cell autoantibodies are persistent, normoglycaemia prevails and there are no symptoms. During stage 2, the number of beta cell autoantibodies may affect the pathogenesis to induce dysglycaemia but there are still no diabetes symptoms. In stage 3, beta cell autoantibodies are still prevalent (some of them may have been lost) and there are symptoms of diabetes. The staging of type 1 diabetes pathogenesis was proposed by Insel et al [1] and the figure is adapted with permission from Insel et al [1]. © 2015 The American Diabetes Association

The aetiology of beta cell autoimmunity is still unclear. Once beta cell autoimmunity has been established, the progression towards clinical type 1 diabetes may be classified into three stages: (1) asymptomatic beta cell autoimmunity with normoglycaemia; (2) asymptomatic beta cell autoimmunity with dysglycaemia and (3) symptomatic type 1 diabetes (Fig. 1) [1]. The sequence of events from autoimmunity to dysglycaemia and then to overt diabetes is predictable but the duration of each phase may vary widely between individuals.

Genetic tests allow risk stratification at birth, although different genes and environmental triggers may influence whether beta cell autoimmunity is marked by insulin autoantibodies (IAA), GAD autoantibodies (GADA), or both, as the first-appearing biomarker [2–4]. A second, third or fourth autoantibody may appear and may influence the progression through each of the stages of the development of type 1 diabetes.

## Genetic aetiology

Type 1 diabetes has a complex genetic background and lacks a clear pattern of inheritance. The disease clusters in some families, yet only about 13% of patients have a first-degree relative with type 1 diabetes [5]. The risk of type 1 diabetes depends on which family members are affected: 3% if the mother, 5% if the father and 8% if a sibling has type 1 diabetes [6]. Having multiple first-degree relatives with type 1 diabetes enhances the risk [7]. Only about half of monozygotic twins are concordant for type 1 diabetes [8], suggesting environmental and/or epigenetic influences [9].

Close to 60 genetic loci have been associated with susceptibility to type 1 diabetes [10]. Variations in the HLA region account for about half of the familial genetic risk, with variants of other genes making smaller individual contributions. It is also worth noting that some genetic factors may be important to the appearance of a first islet autoantibody, reflecting a triggering of beta cell autoimmunity rather than diabetes as such [11].

### The HLA region in type 1 diabetes aetiology and pathogenesis

**Fig. 2:**
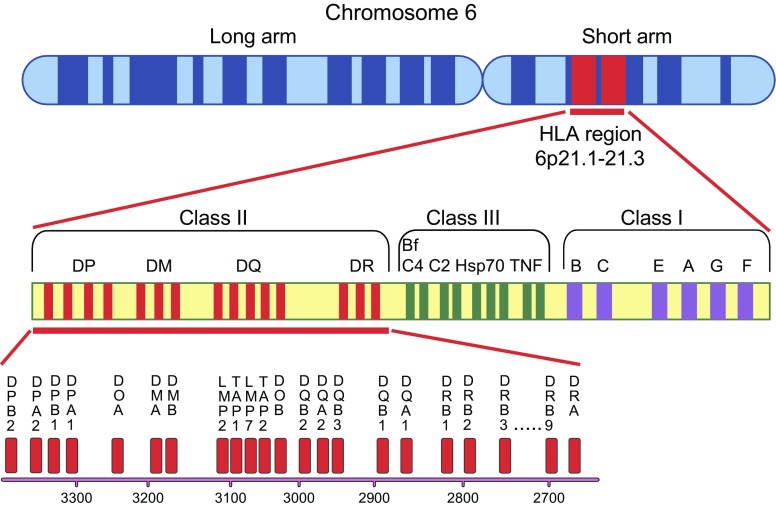
Map of the *HLA DR-DQ-DP* complex region on human chromosome 6, with the class II region shown in greater detail below

The HLA region on chromosome 6p21 is essential to the adaptive immune system. It comprises the class I region at the telomeric boundary, the class II region at the centromeric boundary and the class III region in between (Fig. 2). The HLA region contains over 250 genes, spanning some 4 Mbp, and is the most polymorphic part of the genome [12]. Indeed, polymorphism and pronounced linkage disequilibrium in the HLA region have provided significant challenges in identifying associations between HLA variants and a first-appearing beta cell autoantibody [2, 3].

HLA class II molecules typically present exogenous antigens to T lymphocytes and consist of heterodimers encoded by genes at the *HLA-DR*, *HLA-DQ* and *HLA-DP* loci (Fig. 2). Certain variants in all three loci can influence the risk for a first beta cell autoantibody and type 1 diabetes [2, 3, 13]. Particular combinations of *HLA-DRB1*, *-DQA1* and *-DQB1* alleles can strongly increase or decrease the risk of type 1 diabetes. For example, HLA-DRB1*04 combined with DQA1*03˗DQB1*03:02 confers high risk whereas *HLA*-DRB1*04 combined with DQA1*03-DQB1*03:01 does not [14, 15].

Intuitively, HLA class II heterodimers are important not only to the risk for autoimmune type 1 diabetes as such but also more specifically to both aetiology and pathogenesis (Fig. 1). It is easy to imagine a trigger that is related to a DQ8 heterodimer inducing an autoimmune reaction against proinsulin, reflected in IAA as the first-appearing beta cell autoantibody [2]. Similarly, another trigger may be using the DQ2 heterodimer to induce an autoimmune reaction against GAD65, reflected in GADA [2]. While HLA class II heterodimers are closely related to the aetiology, HLA’s contribution to the pathogenesis cannot be excluded. If beta cell autoimmunity is marked by one beta cell autoantibody only, the risk of progression to clinical onset is low (1:10) [16–18]. The appearance of a second, third or fourth autoantibody, be it IAA, GADA, insulinoma-associated antigen-2 autoantibodies (IA-2A) or zinc transporter 8 autoantibodies (ZnT8A; including autoantibodies against any of the three variants of this transporter, having either W, R or Q at position 325), markedly increases the risk (8:10) [18–20]. Once a first beta cell autoantibody has appeared, the appearance of a second does not seem to be associated with HLA [3]. It is therefore anticipated that *HLA-DR-DQ* will be related to any of the four islet autoantibodies at the time of clinical onset [21–24].

DRB1*03:01-DQA1*05:01-DQB1*02:01 is the most common haplotype, found in 34% and 12.5% of individuals with and without type 1 diabetes, respectively [25]. Children with the high-risk DRB1*03:01-DQA1*05:01-DQB1*02:01/DRB1*04:01-DQA1*03:01-DQB1*03:02 genotype have a 5% incidence of diabetes by 15 years of age [26]. HLA genotypes in the high-risk Scandinavian countries are similar but genetic differences between the countries prevail [27]. In Sweden, the Better Diabetes Diagnosis (BDD) study HLA-typed nearly 4000 individuals newly diagnosed with type 1 diabetes below 18 years of age and found that nine genotypes accounted for 67% of all diabetic individuals compared with 16% of the population (Table 1). All nine genotypes were statistically significantly associated with type 1 diabetes. More importantly, 89% of all 3500 children with type 1 diabetes had at least one copy of either the *DQ8* or the *DQ2* haplotype.

**Table 1:** *HLA-DQ* genotypes conferring risk for type 1 diabetes

Genotype	Diabetic individuals (*n*)	Diabetic individuals (%)	Non-diabetic individuals (%)	OR	*p* value
High-risk HLA					
*DQ2*/*8*	957	27.4	3.5	10.4	<0.00000001
*DQ8*/*8*	355	10.1	1.7	6.7	<0.00000001
*DQ6.4*/*8*	170	4.9	1.2	4.2	<0.00000001
*DQ5.1*/*8*	312	8.9	2.7	3.5	<0.00000001
*DQ4*/*8*	154	4.4	1.4	3.4	<0.00000001
*DQ2*/*2*	167	4.8	1.7	3.0	<0.00000001
*DQ2*/*9*	36	1.0	0.5	2.3	<0.000005
*DQ6.3*/*8*	108	3.1	2.0	1.6	<0.000005
*DQ2*/*6.4*	73	2.1	1.3	1.6	<0.000005
Subtotal	2332	66.7	16.0		
Neutral-risk HLA					
11 genotypes				0.6–1.8	NS
Subtotal	482	13.8	13.0		
Low-risk HLA					
32 genotypes				0.05–1.0	HSNA
Subtotal	468	13.3	46.3		
No-risk HLA					
Many genotypes				NA	
Subtotal	217	6.2	31.8		
Total	3499	100	100		

Details are available in electronic supplementary material Table 1 in [127]

13/32 of these genotypes contained *DQ2* or *DQ8*

HSNA, highly significant negative associations

The importance of the HLA Class II region to the aetiology and pathogenesis of type 1 diabetes calls for high-resolution genetic analyses of the region to be combined with functional assays. The interpretation that type 1 diabetes has two different aetiologies based on a single report [2] requires further studies not only of the genetic aetiology but also of candidate environmental triggers. The notion that one aetiological trigger is associated with *DR4*-*DQ8* and concurrent IAA and the other is associated with *DR3*-*DQ2* and GADA requires better understanding of the HLA region. Once the beta cell autoimmune reaction is established, the pathogenesis includes spreading of the autoimmunity to additional autoantigens. Using next-generation sequencing (NGS), an integrated genotyping system of exons 1–4 was developed to type all alleles of *DRB1*, *DRB3*, *DRB4* and *DRB5* [28, 29] (Fig. 2). NGS typing of children with and without type 1 diabetes revealed that the association between type 1 diabetes and DRB1*03:01:01 was affected by DRB3*01:01:02 and DRB3*02:02:01 [30, 31].

MHC class I molecules present antigens to lymphocytes from within the cell and are encoded at the *HLA-A*, *HLA-B* and *HLA-C* loci. Some MHC class I variants affect the risk of type 1 diabetes, even after adjusting for linkage disequilibrium to MHC class II haplotypes [32]. HLA-B*39:06 was the class I allele most widely associated with type 1 diabetes risk, whereas HLA-B*57:01 decreases the risk [33]. Variation in class I molecules may affect age at diabetes onset rather than the absolute risk of disease [34].

The HLA class III region is genetically dense and codes for several proteins important to the immune system, such as complement factors and TNF-α [12]. Earlier studies of candidate genes provided disparate results [34], although a more recent genome-wide association study reported associations after adjustment for linkage disequilibrium [35]. Recent analyses in The Environmental Determinants of Diabetes in the Young (TEDDY) study, testing whether complement gene SNPs were associated with a first-appearing autoantibody, revealed three SNPs possibly contributing [36].

### Non-HLA genes affecting susceptibility to type 1 diabetes

Non-HLA variants may modify disease risk only in individuals with certain HLA genotypes [37]. Many of these genes are implicated in immune system regulation in particular and beta cell function less often [38, 39]. The non-HLA genes having the strongest influence on type 1 diabetes risk are *INS*, *PTPN22* and *IL2RA* [40].

The association of non-HLA genes with a first-appearing beta cell autoantibody has been tested. The risk for IAA as first autoantibody was associated with *INS*, *SH2B3*, *ERBB3*, *RGS1* and *PTPN22* while GADA as first autoantibody was associated with *CCR7*, *SH2B3*, *TNFAIP3* and *CD226* [11]. Discovering how these genetic factors contribute to the triggering mechanisms of beta cell autoimmunity provides a challenge.

Some genetic susceptibility variants are shared with other autoimmune disorders, including coeliac disease and Crohn’s disease [41]. Known shared loci between type 1 diabetes and rheumatoid arthritis, psoriasis and coeliac disease are concordant, whereas more than half of the SNPs influencing the risk of type 1 diabetes are discordant with genes influencing risk of inflammatory bowel disease and ankylosing spondylitis [42].

*INS* codes for preproinsulin, which is converted by peptidases to proinsulin and insulin. Polymorphisms of *INS* have the strongest association with type 1 diabetes among non-HLA genes [40]. *INS* is unique among susceptibility-enhancing genes as it also codes for a known autoantigen [11].

*PTPN22* encodes a protein tyrosine phosphatase involved in T cell receptor signalling. A gain-of-function mutation proposed to promote autoreactive T cell survival is associated with type 1 diabetes, as well as autoimmune thyroiditis, Crohn’s disease, multiple sclerosis, rheumatoid arthritis and systemic lupus erythematosus [40, 43]. Most established susceptibility genes seem to act multiplicatively with other loci on the risk of disease, except for the joint effect of HLA and *PTPN22* [44]. The minor allele of *PTPN22* is associated with GADA [37].

*IL2RA* encodes IL-2 receptor subunit α, which is expressed on lymphocytes. It has also been associated with multiple sclerosis and systemic lupus erythematosus [40].

## Environmental factors

Environmental factors may trigger either beta cell autoimmunity and the appearance of a first autoantibody [2] or the progression to clinical onset of type 1 diabetes. There is large geographic variation in type 1 diabetes and migrants tend to establish an incidence of diabetes similar to that of the host population [45, 46]. Finland has the highest national incidence of type 1 diabetes (e.g. a hundredfold that of China) [47]. In countries with a lower incidence of type 1 diabetes, the increase in incidence has been more marked [48] whereas in high-prevalence countries it may have decelerated [49]. In parallel with a globally increased prevalence, the proportion of individuals with type 1 diabetes having the high-risk *HLA-DR3/4*-*DQ2/8* genotype has decreased [50].

Infectious agents are among the most extensively studied of the possible environmental triggers. Epidemiological, serological and histological studies, also in experimental animals, support the involvement of viral infections in the aetiology of type 1 diabetes. Proposed mechanisms include T cell cross-reactivity between viruses and islet autoantigens or exposure of beta cell autoantigens to nearby inflammation [51]. Enteroviruses and rubella virus are commonly studied, although the link between congenital rubella infections and type 1 diabetes is controversial [52]. Coxsackie infection during pregnancy may induce beta cell autoimmunity in the mother [53] and may increase the risk for type 1 diabetes in the offspring [54, 55]. Virus studies need to be related to the staging of type 1 diabetes.

A number of nutritional components have been suggested to modulate the risk of type 1 diabetes. Cow’s milk has been associated with both the development of beta cell autoimmunity and progression to type 1 diabetes in children with beta cell autoimmunity [56]. Studies of disease risk in relation to the duration of breastfeeding and the introduction of cereals and solid foods in general to the diet have yielded inconsistent results [45]. Hydrolysed infant formula did not prevent the appearance of beta cell autoantibodies [57].

Observational findings suggest that vitamin D protects against type 1 diabetes by modulating the immune system. However, clinical trials have so far been unsuccessful [45]. Similarly, the anti-inflammatory properties of *n*-3 fatty acids have been suggested to influence the risk of autoimmunity. A prospective birth cohort study found no association between maternal long-chain *n*-3 fatty acid levels during pregnancy and the development of type 1 diabetes in offspring [58]. In one as-yet-unconfirmed report administration of probiotics during the first 27 days of life reduced the risk for a first-appearing beta cell autoantibody in children with the *HLA-DQ2/8* genotype [59].

Factors that increase the body’s requirement for insulin, such as high levels of sugar, may hasten the progression to clinical onset of type 1 diabetes in persons with beta cell autoimmunity [60]. Accordingly, intake of high glycaemic index foods and sugar has been associated with progression to clinical onset in children with beta cell autoantibodies, but not with the development of islet autoimmunity per se [61].

Rapid longitudinal growth, puberty, trauma, low physical activity, being overweight and infections have also been proposed to increase beta cell stress and thereby hasten progression to clinical onset of diabetes in children with beta cell autoimmunity. Psychological stress may increase the probability of both autoimmunity and type 1 diabetes, possibly via increased cortisol, which would increase insulin resistance and directly modulate the immune system [45].

Overall, despite many environmental triggers being implicated in the pathogenesis of beta cell autoimmunity and type 1 diabetes, much of the research appears conflicting. Large, prospective studies of environmental determinants of type 1 diabetes and efforts to translate their findings into preventative interventions are ongoing [62].

## Stage 1: asymptomatic beta cell autoimmunity

### Beta cell autoantibodies

The appearance of beta cell autoantibodies currently represents the earliest established sign of autoimmunity directed towards the pancreatic islet beta cells. Four primary types of islet autoantibodies are detected as markers of beta cell autoimmunity: those against GAD 65, insulin, insulinoma antigen-2 and zinc transporter 8 (which has arginine, glutamine, and tryptophan as variants) [50].

In longitudinal studies of genetically at-risk children followed from birth, beta cell autoantibodies have rarely been detected before the age of 6 months [2]. The peak incidence of the appearance of a first islet autoantibody was at age 9–24 months for IAA and about 36 months for GADA. IA-2A and ZnT8A rarely appeared as a first autoantibody and tended to occur later.

The presence of multiple autoantibodies greatly increases the probability for type 1 diabetes—70% of diabetic individuals have three or four autoantibodies, while only 10% have a single autoantibody [63, 64]. About 96% of individuals are positive for at least one of these four autoantibodies and a number of further candidate autoantibodies have been identified [65].

The duration of stage 1 of type 1 diabetes pathogenesis may vary from a few months to decades. Older age at the appearance of islet autoimmunity, slower progression from single to multiple beta cell autoantibodies and lower IAA titres may predict a delayed clinical onset [66, 67]. Loss of IAA reactivity in children with several autoantibodies was associated with delayed progression to clinical onset [68].

Conversely, in TEDDY, higher IAA and IA-2A titres, but not GADA titres, increased type 1 diabetes in the 5 years that followed the first islet autoantibody [69]. Persons with IA-2A [70, 71] and ZnT8A [71] tend to progress more rapidly to type 1 diabetes than persons without these autoantibodies. Autoantibody titres do not always increase closer to the time of diabetes onset [72]. Up to 60% of persons positive for a single autoantibody may revert to seronegativity [73] and patterns of antibody titres may vary without a clear prognostic significance [74].

A 10 year follow-up of children with multiple islet autoantibodies showed that the *HLA-DR3-DQ2/DR4-DQ8* genotype had a greater risk for diabetes than children with other HLA genotypes [75], yet smaller studies have failed to show that HLA genotype influences the rate of progression [76]. Non-HLA SNPs may influence the rate of progression to clinical onset [66, 76].

### The initiation of beta cell destruction

Beta cell destruction has variously been modelled as a linear or an intermittently relapsing process. The limited human histological evidence available suggests that persistent inflammation of pancreatic islets (insulitis) is present in only a small proportion of non-diabetic, autoantibody-positive individuals and that insulitis emerges closer to the clinical onset [77, 78].

Peripheral CD4^+^ and CD8^+^ T cells with specificity for beta cell autoantigens are often found in persons at high risk of diabetes or in recently diagnosed individuals. Beta cell autoantibodies, long regarded as merely markers of the autoimmune process, may contribute through B cells presenting antigens to CD4^+^ and CD8^+^ T cells [79]. It is thought that cytotoxic T cells, helper T cells, natural killer cells and macrophages contribute to the actual destruction of beta cells [80]. While several modes of action have been proposed in the mouse [81, 82], the pathogenic pathway is less understood in humans. Recent studies of organ donors [77, 83–85] indicate that multiple autoantibodies and an expected proximity to clinical diagnosis was associated with insulitis. Hence, in contrast to the common belief that insulitis needs to precede islet autoantibodies, the latter may be present months to years before mononuclear cells invade the pancreatic islets.

Although B and T cell activity is a known key driver of beta cell destruction, the role of the innate immune system in type 1 diabetes is also recognised [86]. The activation of innate pattern-recognition receptors promotes the synthesis and release of proinflammatory mediators [87]. The relevance of the complement system to type 1 diabetes has been suggested by the association with HLA class III SNPs [35, 36]. Complement component C4d was found in the pancreas of 25% of individuals with type 1 diabetes, 7% of autoantibody-positive non-diabetic individuals and 2% of autoantibody-negative non-diabetic individuals [88].

Current evidence suggests that loss of function and destruction of beta cells begins after the onset of autoimmunity signalled by beta cell autoantibodies. Beta cell destruction may be precipitated by the innate immune system and inflammation, and activated autoreactive lymphocytes are believed to carry out most of the actual beta cell damage. Already in stage 1 of type 1 diabetes, fluctuations of insulin secretion may be detected, probably due to reversible beta cell stressors rather than beta cell death per se [89]. Once functional beta cell mass has declined sufficiently, an individual’s blood glucose begins to deviate. The individual thus enters stage 2 of the progression towards manifest type 1 diabetes.

## Stage 2: asymptomatic beta cell autoimmunity with dysglycaemia

At this stage, enough functional beta cell mass has been lost that biochemical tests may reveal impaired glucose tolerance. As yet, no symptoms develop.

Repeated, longitudinal GTTs in persons progressing towards clinical onset show a pattern of altered insulin and C-peptide secretion and reduced glucose clearance as beta cell mass declines [90]. An early sign of beta cell failure is a reduced early-phase insulin response during an IVGTT or OGTT [91].

During the decline of first-phase insulin and C-peptide responses, the late-phase insulin response (defined as insulin secretion after 30 min) increases, so that the total AUCs for insulin and C-peptide over time remain essentially stable throughout much of the prodromal period. The maximal glucose excursions and the duration of elevated postprandial blood glucose during an OGTT increase as overt diabetes approaches [90]. Deterioration of insulin response measured as peak C-peptide and the 2 h glucose value during an OGTT seem to accelerate in the months before diabetes diagnosis [92, 93].

Decreasing glucose tolerance is often reflected by a gradually increasing HbA_1c_ within the reference range. Increase in HbA_1c_ may be used as a marker of high specificity but poor sensitivity for clinical onset [94]. Longitudinal measurements of random glucose values are less useful [95]. Markers such as fructosamine, 1,5-anhydroglucitol and glycated albumin reflect glycaemic control on a timescale intermediate to those of daily glucose and HbA_1c_ and could therefore potentially increase the sensitivity of beta cell function compared with HbA_1c_. All three markers are predictive of type 2 diabetes [96] but their utility for predicting type 1 diabetes has not been tested.

## Stage 3: symptomatic type 1 diabetes

At the clinical onset of type 1 diabetes, remaining beta cells produce insufficient insulin to prevent persistent hyperglycaemia, with its classic symptoms of polyuria, polydipsia and polyphagia. Autopsy studies show a beta cell mass of about 10% of normal in individuals with recent-onset type 1 diabetes. However, as these studies are performed mostly in persons who died of diabetic ketoacidosis, this probably underestimates the remaining beta cell mass in most newly diagnosed individuals [97]. Indeed, those who are older at onset tend to have more beta cells than young children and loss of 40% of beta cells can be sufficient to induce symptoms in a 20-year-old person [98].

In agreement with these findings, one-third of young people maintain C-peptide levels within the reference range 1 year after diagnosis [99]. Children with higher preserved C-peptide levels tend to be older at diagnosis and have a higher age-adjusted BMI. When interpreting these data, one should bear in mind that participants in longitudinal investigations of at-risk individuals tend to be diagnosed with type 1 diabetes earlier in the disease course than individuals in the general population and therefore tend to have higher C-peptide levels [100].

Following the initiation of insulin treatment, about 80% of children and adolescents experience partial remission, with reduced insulin requirements. This is attributed to transiently improved insulin secretion and peripheral insulin sensitivity [50]. It is believed that the treatment of hyperglycaemia reverses beta cell exhaustion [101]. In the Diabetes Control and Complications Trial, aggressive treatment of hyperglycaemia was associated with preserved beta cell function [102]. In contrast, a more recent study failed to demonstrate preserved beta cell function in newly diagnosed individuals receiving intensified anti-hyperglycaemic therapy compared with those receiving current standards of care [103].

Retained beta cell function is associated with lower HbA_1c_ values and reduced probability of both long-term diabetic complications and severe hypoglycaemia [100]. Recent studies employing highly sensitive C-peptide assays suggest that individuals with long-term type 1 diabetes secrete residual amounts of insulin [104]. This is consistent with histological evidence that functional beta cells can be found in long-standing type 1 diabetes [105]. Insulitis may persist for years after diabetes onset but correlates negatively with duration of diabetes [78]. Ongoing insulitis seems to correlate with remaining beta cells, whereas persons without histological evidence of insulitis rarely have residual amounts of insulin [78].

Despite the presence of beta cells in some individuals with diabetes of long duration, the insulin response to OGTT deteriorates further after type 1 diabetes becomes symptomatic, suggesting continued beta cell loss. C-peptide response is reduced overall and glucose levels are elevated at 60, 90 and 120 min, but not at 30 min, in children during repeat OGTTs within 3 months of an initial positive diagnostic OGTT [92]. Temporary remission following diagnosis notwithstanding, patients soon become dependent on exogenous insulin for survival.

## Novel biomarkers with potential to allow early prediction

Emerging novel technologies provide opportunities for uncovering biomarkers that may predict beta cell autoimmunity and type 1 diabetes. These include transcriptomics, proteomics, metabolomics, intestinal microbiome DNA sequencing and beta cell-derived proteins and nucleic acids. In addition to biomarkers for the prediction of type 1 diabetes itself, predictors of angiopathic and neuropathic complications of the disease are being investigated [106].

Transcriptomics (study of patterns of gene expression) has shown promise for risk stratification of autoantibody-positive individuals [107]. It has been reported that a proinflammatory signature of gene expression is present both in individuals with recent-onset type 1 diabetes and in high-risk individuals who later progress to diabetes. In the latter group, the gene expression signature preceded the appearance of autoantibodies [108, 109]. These studies point towards dysregulation of the innate immune system having potential as an early predictor of adaptive beta cell autoimmunity.

Proteomic studies have also suggested that patterns of immune dysregulation characterise type 1 diabetes [110]. Still, studies are few and much work remains to be done before proteomic hallmarks predictive of beta cell autoimmunity and type 1 diabetes are established.

Metabolomics techniques have shown that persons who progress to diabetes have different levels of certain lipids when compared with persons who remain non-diabetic [111]. There is evidence that these differences exist already in utero; altered lipid content of the umbilical cord may reflect a pathogenic pregnancy and an increased chance of developing type 1 diabetes at an earlier age. Cord blood phosphatidylcholines and phosphatidylethanolamines were significantly decreased in children diagnosed with type 1 diabetes before 4 years of age [112, 113].

In a longitudinal study, serum metabolite profiles were compared between children who were autoantibody-negative and remained healthy and those who progressed to clinical onset of type 1 diabetes [111]. The latter children showed already-reduced serum levels of succinic acid and phosphatidylcholine in the cord blood while triacylglycerols and antioxidant ether phospholipids were reduced during follow-up and proinflammatory lysophosphatidylcholines increased several months before the appearance of a first islet autoantibody. Specifically, before the appearance of IAA and GADA there was a decrease in ketoleucine and an increase in glutamic acid. Therefore, an early metabolic change preceding autoimmunity cannot be ruled out. At the time of clinical onset only specific phospholipids remained diminished as compared with matched non-progressors. The authors concluded that a reduction in choline-containing phospholipids in cord blood is associated with progression to type 1 diabetes but not with development of beta cell autoimmunity [111].

The human gut microbiome has altered significantly during the last century in response to changes in nutrition and the use of antibiotics, among other environmental factors. Studies so far suggest that the diversity of intestinal bacterial flora is reduced in autoantibody-positive persons who progress to type 1 diabetes. Alterations of the microbiome have not yet been demonstrated before the appearance of autoantibodies. The current evidence thus suggests that the microbiome may reflect the development of clinical disease in persons with demonstrable autoimmunity, but not the initiation of autoimmunity itself [114]. Mechanistically, it has been suggested that the microbiome affects innate immunity and may be linked to inflammatory serum signatures [87]. Studies on the role of the microbiome in type 1 diabetes are still at an early stage of development and have so far been observational.

Markers of T cell activation and beta cell destruction (such as beta cell-specific DNA, RNA and proteins) have been studied as indicators of increased diabetes risk and diabetes [115]. Nanoparticles have been used in MRI to visualise insulitis in individuals with recent-onset type 1 diabetes [116], and further studies of non-invasive radiological methods for insulitis are of interest.

Scores combining biochemical and clinical characteristics to predict the risk of type 1 diabetes have been derived from trials of high-risk individuals [117–119]. The Diabetes Prevention Trial Risk Score was developed through the Diabetes Prevention Trial 1, which included islet autoantibody-positive relatives of individuals with type 1 diabetes. The model was later tested in the TrialNet Natural History, in which 2 and 3 year chance for diabetes was found to be similar to that in the original cohort [120]. The models have not yet been validated in a general population.

‘Omics’ techniques and other novel biomarkers are still in their infancy for predicting the stages of type 1 diabetes. Validation studies across multiple, large cohorts are needed. Identifying persons in stages that precede beta cell autoimmunity and understanding the mechanisms of early stages may offer new opportunities for preventative interventions (Table 2).

**Table 2:** Established and emerging predictors of type 1 diabetes

Predictor	Risk factor	Detectable before diagnosis	Reference
HLA genotype	Certain HLA class II haplotypes; to a lesser extent, class I haplotypes	From gestation	[13, 33]
Non-HLA genotype	About 50 candidate genes	From gestation	[37]
Beta cell-derived proteins and nucleic acids	Increased unmethylated *INS* DNA reflects beta cell death	Months to years	[89]
Serum transcriptome	Proinflammatory pattern of gene expression	Months to years	[108]
Serum proteome	Patterns of immune activation?	Unknown	[110]
Serum metabolome	Reduced phosphatidylcholine	From gestation	[113]
Gut microbiome	Decreased bacterial diversity	Unknown	[114]
Autoantibodies	Risk increases with number of autoantibody types	Months to decades	[50]
Minor autoantibodies	Found in fewer than 30% of individuals	Unknown	[123]
HbA_1c_	Increase from baseline	Months to years	[94]
IVGTT/OGTT	Reduced early-phase insulin response and elevated postprandial glucose	Months to years	[90]
Radiology	Pancreatic nanoparticle uptake indicating insulitis	Unknown	[116]

## Current gaps in knowledge and possible future directions

Despite significant advances in understanding type 1 diabetes, our grasp of the aetiology and pathogenesis and our ability to predict the disease is incomplete, as outlined below.

Variants of some 50 genes have been identified as affecting the risk for type 1 diabetes [121]. Research investigating how these genetic variants affect the progression through each of the three stages of type 1 diabetes is needed. Interactions between different genes, between genes and the environment and between different environmental factors need investigating.

A major question is whether individuals destined to develop type 1 diabetes can be identified before the appearance of beta cell autoantibodies. Robust prediction of autoantibodies would seem an opportunity for new preventative strategies. Primary prevention with oral insulin has been attempted in newborns [122].

A number of minor autoantigens have been implicated in the pathogenesis of type 1 diabetes. Autoantigens are regarded as ‘minor’ when less than 30% of newly diagnosed type 1 diabetes persons exhibit autoantibodies. Many of these autoantibodies, found in the general population, are present in a higher proportion of persons with type 1 diabetes [123]. The natural history of minor autoantigens in type 1 diabetes, their contribution to the development of disease and their predictive value remains largely obscure.

A small proportion of individuals with type 1 diabetes are negative for all the major islet autoantibodies [124]. Further research needs to clarify whether these people were previously autoantibody-positive but then converted to seronegativity, whether they have an immune response against hitherto unidentified beta cell antigens or known minor autoantigens, or whether their condition represents a type of diabetes with an aetiology altogether different from that of type 1 diabetes.

Less is known about new-onset type 1 diabetes in adults than in children. The incidence is difficult to ascertain since many adults with type 1 diabetes will be misclassified as type 2 diabetes. In adults, GADA appear more frequently than other islet autoantibodies and symptoms following progressive insulin deficiency may appear more gradually [125, 126].

## Summary and conclusions

In summary, genetic variation in the HLA region is the major determinant of diabetes risk at birth. It is now possible to study not only the genetic but also the environmental aetiology that may involve triggers causing the appearance of a first islet autoantibody, be it IAA in children with *HLA DR4-DQ8* or GADA in children with *DR3-DQ2*. The mechanisms of the long-sought-after association between HLA and type 1 diabetes needs to be evaluated through the mechanisms of antigen presentation by HLA class II heterodimers. High-resolution sequencing and detailed mapping of the HLA region will be important to disclose gene–environmental interactions. The appearance of multiple islet autoantibodies strongly predicts the clinical onset of type 1 diabetes. In persons with multiple islet autoantibodies, progressive dysglycaemia signals increasing proximity to overt hyperglycaemia and clinical onset. It is likely that the three-stage model of type 1 diabetes will be updated and improved as beta cell autoimmunity and beta cell loss of function and destruction become better understood. Further risk stratification will allow the identification of individuals predestined to type 1 diabetes to allow preventative measures. Refined predictors of islet autoimmunity and type 1 diabetes may ultimately enable successful interventions to prevent the disease altogether.

## Electronic supplementary material

ESM downloadable slideset (PPTX 260 kb)

## Abbreviations

GADA: GAD autoantibodies
IA-2A: Insulinoma-associated antigen-2 autoantibodies
IAA: Insulin autoantibodies
NGS: Next-generation sequencing
TEDDY: The Environmental Determinants of Diabetes in the Young
ZnT8A: Zinc transporter 8 autoantibodies
