# Supplementary material for: Early prediction of autoimmune (type 1) diabetes
Source: Diabetologia. 2017 May 26;60(8):1370–81. doi: 10.1007/s00125-017-4308-1 (PMC5491594; doi:10.1007/s00125-017-4308-1)
Supplement: Supplementary file 1 — (PPTX 260 kb) [file 125_2017_4308_MOESM1_ESM.pptx]

## Slide 1
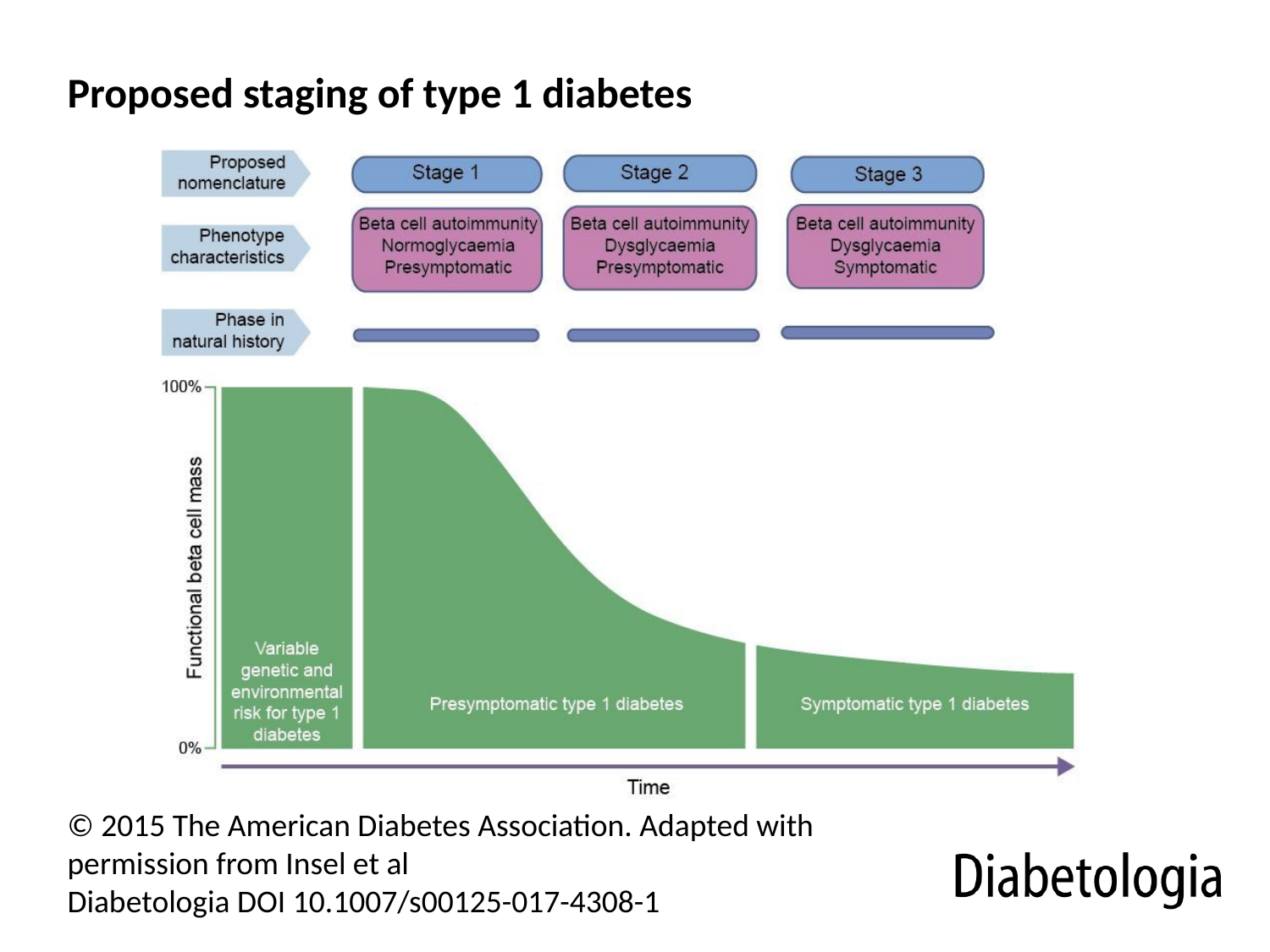

Proposed staging of type 1 diabetes
# Insert figure
© 2015 The American Diabetes Association. Adapted with permission from Insel et al
Diabetologia DOI 10.1007/s00125-017-4308-1

## Slide 2
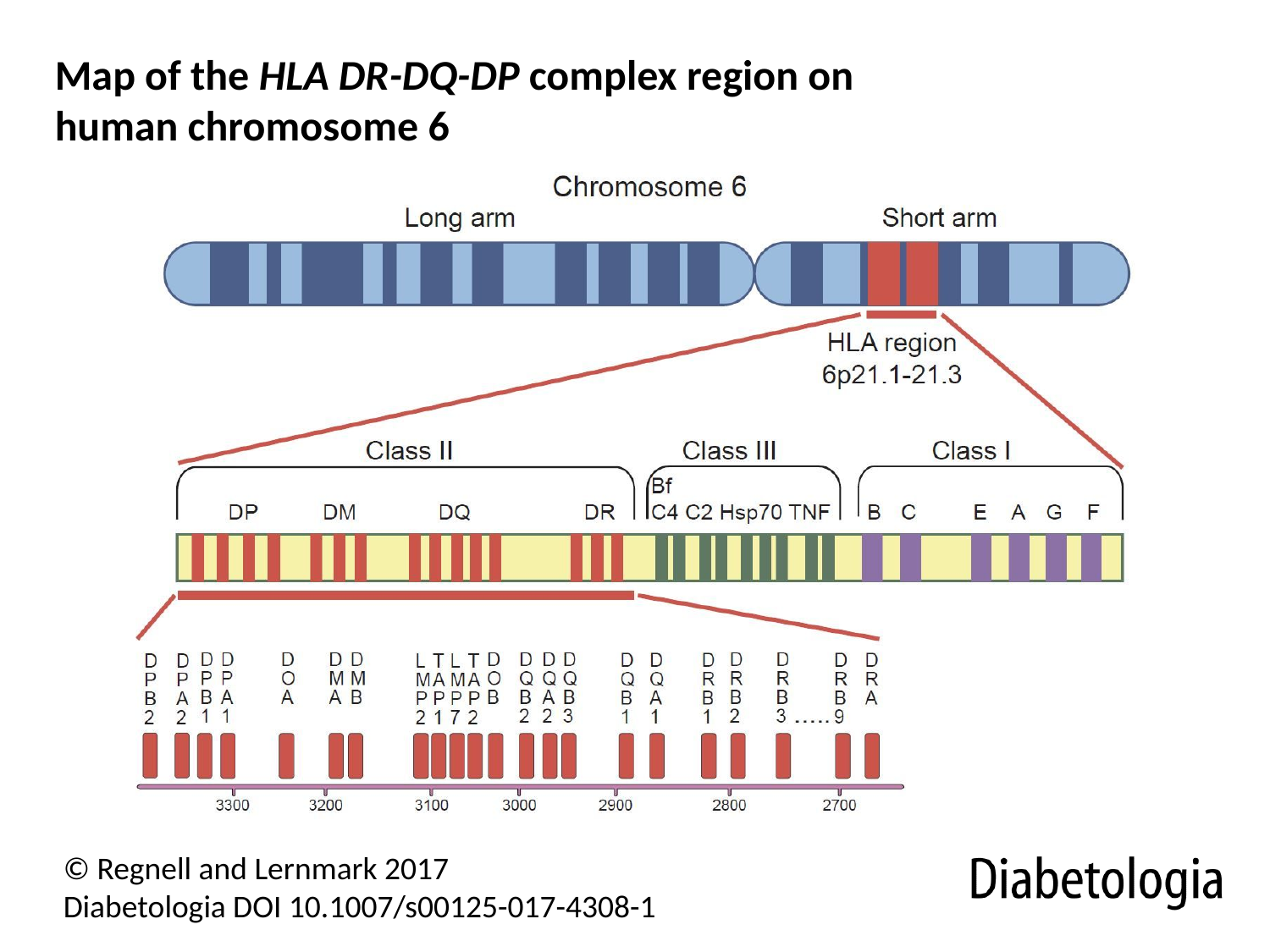

Map of the HLA DR-DQ-DP complex region on human chromosome 6
# Insert figure
© Regnell and Lernmark 2017
Diabetologia DOI 10.1007/s00125-017-4308-1
